# A descriptive study of Swedish women with symptoms of breast inflammation during lactation and their perceptions of the quality of care given at a breastfeeding clinic

**DOI:** 10.1186/1746-4358-2-2

**Published:** 2007-01-23

**Authors:** Linda J Kvist, Marie Louise Hall- Lord, Bodil Wilde Larsson

**Affiliations:** 1 Department of Obstetrics and Gynaecology, Hospital of Helsingborg, SE-251 87, Sweden; 2 Department of Nursing, Karlstad University, SE-651 88, Sweden; 3 Department of Nursing, Gjorvik University College, Norway

## Abstract

**Background:**

Women's perceptions of quality of care during episodes of breast inflammation have been scantily explored. It was the objective of the present study to describe a cohort of breastfeeding women with inflammatory symptoms of the breast during lactation regarding demographical variables, illness history and symptoms at first contact with a breastfeeding clinic and to explore their physical health status, psychological well-being and perceptions of quality of care received, at a six-week postal follow-up.

**Methods:**

This is a descriptive study set at a midwife-led breastfeeding clinic in Sweden, which included a cohort of women with 210 episodes of breast inflammation. The women had taken part in a RCT of acupuncture and care interventions and were recruited between 2002 and 2004. Of the total cohort, 176 (84 %) responded to a postal questionnaire, six weeks after recovery.

**Results:**

Of the 154 women for whom body temperature was recorded at the first visit, 80 (52%) had fever ranging from 38.1°C to 40.7°C. There was no significant difference between those with favourable outcomes (5 or less contact days) and those with less favourable outcomes (6 or more contact days) for having fever or no fever at first contact. Thirty-six percent of women had damaged nipples. Significantly more women with a less favourable outcome (6 or more contact days) had damaged nipples. Most women recovered well from the episode of breast inflammation and 96% considered their physical health and 97% their psychological well-being, to be good, six weeks after the episode. Those whose illness lasted 6 days or more showed less confidence in the midwives and in the care given to them. Twenty-one (12%) women contacted health care services because of recurring symptoms and eight of the 176 responders (4.5%) were prescribed antibiotics for these recurring symptoms. A further 46 women (26% of the responders) reported recurring symptoms that they managed without recourse to health care services.

**Conclusion:**

Initial fever may not be indicative of outcomes for women with inflammatory breast symptoms and treatment by antibiotic therapy may be necessary less often than has been supposed. Women who are also suffering from damaged nipples may need special attention. Those with protracted symptoms were less satisfied with care and showed less confidence in caregivers. International research collaboration might help us find the optimal level of antibiotic therapy for this group of women. This is an important consideration for the global community.

## Background

During the last decade, researchers have worked to gain an understanding of the disease processes involved in mastitis during lactation [1-3]. Human breast milk contains protective factors such as antioxidants, lactoferrin, leukocytes and antagonists for pro-inflammatory cytokines [4-6]. It appears that the special composition of human breast milk and the conditions within the lactating breast may render this inflammation dissimilar to inflammation occurring in other parts of the body and in other circumstances [4,5]. The context within which a new mother finds herself feeling extremely ill is also special. The demands of new motherhood may weigh heavily [7] when faced with extreme fever, excruciatingly painful breasts and an infant who wants nothing more than to suckle [8]. Most previous studies of inflammatory symptoms of the breast during lactation have focused on risk factors and mechanisms involved in the development and resolution of the disease [1,2,9-11].

The present study was nested within a randomised controlled trial (RCT) of care interventions and acupuncture for the treatment of breast inflammation during lactation which was carried out at a breastfeeding clinic in the south of Sweden [12]. It was considered that the outcome of an episode of breast inflammation, as measured by number of contact days with the breastfeeding clinic, might be affected by other variables than the care interventions used. Patients' perceptions of the quality of care they receive are an important aspect of health care evaluation [13] and are increasingly used to inform and improve clinical care practices [14]. Factors such as confidence in health care providers and belief in their methods of treatment may also be involved in a return to health [15]. A study from the USA in 2003 described the symptoms, self-care, treatment, burden of mastitis, recurrence of symptoms and complications experienced by 31 breastfeeding women [16]. Wambach found that continued breastfeeding was the self-care practice most often used and symptoms affected activities of daily living more than they affected breastfeeding [16]. At six weeks after the episode of mastitis, 19% had experienced recurrent symptoms [16].

No earlier study has been found where women's perceptions of quality of care in relation to episodes of breast inflammation have been explored. It was therefore the objective of the present study to describe a cohort of breastfeeding women with inflammatory symptoms of the breast during lactation with regards to demographical variables, illness history and symptoms at first contact with a breastfeeding clinic and to explore their physical health status, psychological well-being and their perceptions of quality of care received, at a six week postal follow-up.

## Methods

### Sample

The cohort consisted of women who were recruited to a RCT of acupuncture when they made contact with a midwife-led breastfeeding clinic because of symptoms of breast inflammation during a current lactation [12]. During the study period child welfare clinics in the uptake area were asked to refer all women with inflammatory breast symptoms to the breastfeeding clinic. After the study was completed the welfare clinics were asked to give account of how many women they had treated for breast inflammation. One report was made by one clinic. The emergency service in the uptake area for obstetrical and gynecological problems is part of the same clinic where the study took place and women with breast inflammation were therefore easily referred to the breastfeeding clinic. During the two year period of the study, 291 women contacted the clinic because of inflammatory symptoms of the breast. Of these, 46 declined participation in the study because of lack of time or fear of needles. Eleven women did not have sufficient Swedish language skills, one was unsuitable for acupuncture because of a skin disease and five had started treatment elsewhere, prior to their visit to the clinic. For a further 18, no reason for non-participation was recorded.

The symptoms could be any mixture of erythema, increased breast tension that was not relieved by breastfeeding, fever, pain and lumps in the breast tissue. It was explained to the women that whether they participated or not they would be treated and followed up until they felt well enough to discontinue contact with the breastfeeding clinic. A woman was considered as having a new episode of breast inflammation, and randomised again, when more than six weeks had elapsed since her latest contact with the breastfeeding clinic. A total of 205 women who had suffered from 210 episodes of breast inflammation were recruited and randomised to one of three treatment groups.

### The treatment groups

In Sweden one of the mainstays of treatment for inflammatory symptoms has for many years been oxytocin nasal spray given at the discretion of the midwife. The rationale for this treatment is that oxytocin helps the milk ejection reflex, which may have been effected by over-distension of the breast. Before the start of the RCT two experts in acupuncture (one midwife and one obstetrician) suggested that for those receiving acupuncture treatment no oxytocin spray should be given. The reason for this was that one of the acupuncture points chosen, SP (spleen) 6, is considered to have an oxytocin-like effect [17]. One of the treatment groups was given neither acupuncture at point SP 6 nor oxytocin spray. All the three treatment groups were given advice about interval and duration of breastfeeds and about emptying of the breasts by manual expression, pumping or warm shower to the breasts. This is referred to as essential care. Thus the groups were as follows:

*Group 1: *essential care and the use of oxytocin spray as deemed necessary by the midwife;

*Group 2: *essential care and treatment by acupuncture needles placed at HT (heart) 3 and GB (gallbladder) 21;

*Group 3: *essential care and treatment by acupuncture needles placed at HT 3, GB 21 and SP 6.

Of the total study population of 210 randomisations, 31 (15%) were prescribed antibiotic therapy in the RCT. Of the 210 randomisations made 176 (84%) women completed and returned a questionnaire sent to them six weeks after their illness. Of these 176, 54% were primiparous and 46% multiparous.

In the RCT, scores for breast erythema, increased breast tension that was not relieved by breastfeeding and pain were measured on scales that were added together to create a Severity Index. The scales were tested using Cronbach's alpha and the alpha scores were 0.79, 0.82 and 0.81 on contact days 3, 4 and 5 respectively. Analyses showed that there were no statistically significant differences between the treatment groups for length of contact with the breastfeeding clinic, for numbers of women requiring antibiotic treatment or for numbers of women on contact days 3, 4 and 5 with the lowest possible score for severity of symptoms [12]. The non-acupuncture group had significantly higher scores for mean Severity Index than the two acupuncture groups on days 3 and 4, p = 0.01. More women with damaged nipples were given antibiotics than those without nipple damage, but this difference was not statistically significant, p = 0.09 [12].

### Instruments

#### Data collection sheets

Once the woman had been randomised to one of the three treatment groups, data collection sheets were filled in which included background variables, history of the illness and the woman's symptoms at first contact. These included whether the woman had damaged nipples and whether the damage was in the form of a sore or fissure and where on the nipple this damage occurred: at the tip of the nipple, across the top of the nipple or round the base. A 5-point scale was used to measure breast erythema: 0 = no redness and 4 = bright red over most of the breast. Breast tension was measured on a 6-point scale: 0 = soft, no change and 5 = very tense and very painful. Women's pain was measured on a visual analogue scale (VAS) where 0 = no pain and 10 = worst possible pain. The scale was graduated with numbers from 0 to 10. The woman was shown the scale and asked to indicate where on the scale she judged her pain to be. The woman or the midwife then drew a cross in the place where the woman indicated.

#### Questionnaire

A questionnaire, *Quality from the Patient's Perspective (QPP)*, has been developed in Sweden by Wilde et al [18,19]. From analyses of qualitative and quantitative data Wilde's work resulted in a model with four quality dimensions that can explain the patients' perspectives on quality of health care. This model was used to develop questionnaires that have been internationally tested and validated [20]. A study-specific questionnaire was used in this study and consisted of 17 items, 12 of which were inspired by the identity-oriented approach dimension of the *QPP *questionnaire.

The women were asked to rate their physical health and psychological well-being on 5-point scales: 1 = very poor to 5 = very good. Opinions about being given the best possible advice and instructions and confidence in the midwife and/or doctor who gave the advice were all measured on 3-point scales: not at all, partly or completely. Twelve of the questions were answered on two 4-point response scales for each item. The first measures perceived reality (PR) of quality of care and the second measures the subjective importance (SI) of the item to the individual. The PR scale ranges from "do not agree at all" to "fully agree" and the SI scale from "of little or no importance" to "of very great importance". Recurrent symptoms within six weeks were also explored. Women were asked how many days after recovery from their first episode the new symptoms had started.

### Data collection

The data were collected at a midwife-led breastfeeding clinic in the south of Sweden between 2002 and 2004. Women were invited to join the RCT when they made contact with the clinic. The midwives filled in data collection sheets together with the participating women at the first visit to the clinic. These collection sheets included measurements of erythema, breast tension and pain. Daily telephone contacts were made with each mother and the mother was asked to rate her symptoms (erythema, increased breast tension and pain) on the scales that she had filled in together with the midwife and in relation to how her symptoms had been on the previous day. Daily telephone contacts were maintained until the woman and the midwife were in agreement that contacts could be terminated. The woman should feel herself to be on the way to recovery. The date of this decision was noted and six weeks later a follow-up questionnaire was posted to each woman by the first author. Reminders, with another questionnaire were sent after two weeks and pre-paid, addressed envelopes were provided. As far as was possible the same midwives contacted the women that they had met at the breastfeeding clinic. Sometimes it was not possible for the same midwife to contact the woman because of off-duty times and illness. In these cases another midwife telephoned the mother.

### Ethical considerations

On arrival at the clinic the women were given verbal information by the midwife and also written information informing them of their right to refuse without any detriment to their care and also the right to leave the study whenever they wished. The women were informed that if they joined the study they would be asked to answer a questionnaire which would be posted to them six weeks after their recovery. The committee for medical research ethics in Lund, Sweden approved the study, protocol number LU 592-00.

### Data analysis

The material was analysed using *SPSS *version 14.0 (SPSS, Inc., Chicago, USA). The mean number of contact days needed with the breastfeeding clinic until the woman felt well enough to discontinue contact was 5 (SD = 2.9). A favourable outcome was considered as those with ≤ 5 contact days and a less favourable outcome as those with ≥ 6 contact days. The Mann Whitney U-test was used to test whether the mean number of days women had experienced symptoms before contact with the breastfeeding clinic was different between the favourable and less favourable outcome groups. Odds ratios (OR) with 95% confidence intervals (CI) were calculated for proportions of women in the favourable/less favourable groups who had any fever *versus *no fever, damaged nipples *versus *no nipple damage and unilateral *versus *bilateral symptoms. Mean scores for pain, increased breast tension not relieved by breastfeeding and erythema were compared between the outcome groups using the one-way ANOVA.

The one-way ANOVA test was also used to test differences in means scores for the answers to the questionnaire between the three treatment groups in the RCT. The Mann Whitney U-test was used to test the mean scores for items in the questionnaire between the following groups: favourable outcome (n = 126) and less favourable outcome (n = 50), standard education (basic plus upper secondary, n = 104) and higher education (n = 98), primiparous (n = 103) and multiparous (n = 104) women, recurrent symptoms (n = 67) and no recurrent symptoms (n = 109). Responders and non-responders were compared for maternal age, nationality, parity, severity of symptoms at first contact, self-reported physical health and psychological well-being at first contact and number of contact days to recovery. The tests were two-tailed and significance was accepted as p = ≤0.05.

## Results

### The cohort

The cohort consisted of 205 women with 210 episodes of inflammatory symptoms of the breast. Table 1 shows a summary of background variables. The majority of the women were Swedish nationals (95.7%) and 51.5% had received standard education and 46.7% had attended college or university. There was equal distribution between primiparous and multiparous women. The mean maternal age was 31 yrs (SD = 4.6). The women's symptoms occurred between 1–76 weeks postpartum, with a median of 3 weeks. More than half (58.6%) of the women experienced symptoms during the first 4 postpartum weeks. A total of 8 women had undergone previous breast surgery, 2 augmentation surgeries, 5 reductions and 1 unknown type. Pacifiers were used by 44.4% of the babies and nipple shields by 15.9% of the women.

**Table 1:** Background variables at women's first visit to the breastfeeding clinic

	**Total study population** **(*n *= 210)** ***n *(%)**	**Follow-up responders** **(*n *= 176)** ***n *(%)**
**Nationality**		
Swedish	199 (95.7)	167 (94.8)
Scandinavian	1 (0.5)	1 (0.6)
Other	10 (3.8)	8 (4.5)
**Education**		
Basic	8 (4.0)	8 (4.5)
Upper secondary school	96 (47.5)	80 (45.5)
College/University	98 (46.7) 8 missing	83 (47.2) 5 missing
**Parity**		
Primiparous	103 (49.0)	94 (53.4)
Multiparous	104 (49.5) 3 missing	80 (45.5) 2 missing
**Previous breast surgery**		
Reduction	5 (2.4)	5 (2.8)
Augmentation	2 (1.0)	2 (1.1)
Not recorded	1 (0.5)	-
**Weeks postpartum at first symptoms**		
1 – 4 weeks	123 (58.6)	105 (59.7)
4.1 – 8 weeks	36 (17.1)	30 (17.5)
8.1 – 12 weeks	10 (4.8)	10 (5.3)
12.1 – 16 weeks	15 (7.1)	13 (7.2)
> 16 weeks	26 (12.4)	18 (10.3)
**Using a nipple shield**		
Yes	33 (15.9)	28 (15.9)
No	175 (85.6) 2 missing	146 (83.0) 2 missing
**Baby using pacifier**		
Yes	92 (44.4)	72 (42.6)
No	115 (55.6) 3 missing	98 (55.7) 3 missing
**Multiparous women who had breast inflammation with previous child**		
Yes	47 (45.2)	
No	48 (46.2) 9 missing	

The mean number of contact days needed until the mother and midwife agreed to discontinue contact was 5.0 (SD = 2.9). Table 2 shows the symptoms experienced by women with favourable (≤ 5 contact days) and less favourable outcomes (≥ 6 contact days), at their first meeting with the midwife at the breastfeeding clinic. The difference between the outcome groups for any fever *vs *no fever was not significant; OR 0.50 (95%CI 0.17, 1.10). The percentage of women with damaged nipples was 36%. There were significantly more women in the less favourable outcome group who had damaged nipples: OR = 2.70 (95%CI 1.40, 5.14), p < 0.01. Unilateral symptoms were most common (85.6%) and there was no significant difference between proportions in the outcome groups with bilateral symptoms. The length of time women had symptoms of breast inflammation before contacting the clinic ranged from less than 24 hours to 7 days. There was a difference between the favourable and less favourable outcome groups for the number of days symptoms were apparent before contact was made, but this was not significant (p = 0.07). Women with a less favourable outcome had significantly higher means scores for increased breast tension that was not relieved by breastfeeding, p < 0.01, and for erythema, p < 0.01. There was no significant difference in the women's experiences of pain measured on the VAS. The midwives asked the doctor to examine 6 (2.9%) women at the first visit.

**Table 2:** Symptoms and history of the illness for women with a favourable outcome (≤ 5 contact days) and a less favourable outcome (≥ 6 contact days) at their first visit to the midwife

	**Women with a favourable outcome (≤ 5 contact days) *n *= 149**	**Women with a less favourable outcome (≥ 6 contact days) *n *= 61**	**Total missing values**	**Test ** (significance level ≤ 0.05)
	***n (%)***	***n (%)***		

**Fever**				
No fever (≤ 37.5°C)	34 (22.8)	9 (14.8)		Any fever *vs *no fever OR 0.50 (95%CI 0.17, 1.10) NS*
Moderate fever (37.6 – 38°C)	19 (12.8)	12 (20.0)		
Severe fever (38.1 – 40.7°C)	51 (34)	29 (47.5)	56	
**Nipple damage**				
No damage	99 (66.4)	28 (45.9)	6	Any damage *vs *no damage OR 2.70 (95%CI 1.40, 5.14) *p *= < 0.01
Damage at end of nipple	30 (20.1)	22 (36.1)		
Fissure across nipple	2 (1.3)	7 (11.5)		
Fissure around base of nipple	12 (8.1)	4 (6.6)		
**Unilateral involvement**	131 (88.0)	48 (79.1)		
**Bilateral involvement**	16 (10.7)	13 (21.3)	2	Uni *vs *bilateral OR 2.22 (95%CI 0.90, 5.32) NS

	**Mean (± SD)**	**Mean (± SD)**		

**Number of days illness before contacting clinic**	2.6 (2.1)	3.1 (2.8)		Mann Whitney z = -1.77 NS
**Breast pain on VAS at first contact (range 0 – 10)**	5.5 (2.4)	6.0 (2.0)		ANOVA F = 2.52 NS
**Increased breast tension at first contact (range 0 – 5)**	3.1 (1.5)	3.9 (1.0)		ANOVA F = 16.70 *p *= < 0.01
**Breast erythema at first contact (range 0 – 4)**	1.7 (1.1)	2.2 (1.2)		ANOVA F = 9.30 *p *= < 0.01

*NS = not significant

During the study period there were 5,225 births at the regional hospital and 92% of babies in the uptake area were breastfed for at least two months. Since a large majority (75%) of women experienced their symptoms within these two months it may be estimated that the total breastfeeding population at the time of the study was approximately 4,807 women. A total of 291 women contacted the clinic because of breast inflammation. Thus, we estimate that in this population the incidence was approximately 6%. The use of a nipple shield increased the risk for a less favourable outcome: OR 3.20, (95%CI 1.46, 7.30). The use of a pacifier for the baby did not affect the odds of a less favourable outcome: OR 0.90, (95%CI 0.44, 1.63). A total of 31 women (15% of the 210 cases) were prescribed antibiotics. Seven women (3.3% of the 210 cases and 0.1% of 4,807 breastfeeding women) were treated for breast abscess during the RCT [12]. None of the women stopped feeding their baby from the breast during the episode of inflammation and no adverse effects to the infants of continued breastfeeding were reported.

### Follow-up questionnaire

The 12 questions that measured women's views on the quality of care received were tested for reliability using Cronbach's alpha. The alpha score for perceived reality was 0.73 and for subjective importance it was 0.75. One hundred and seventy-six (84%) of the 210 questionnaires that were sent out were returned. There was a significant difference between responders and non-responders for parity; significantly more of the non-responders were multiparous women (chi^2 ^= 6.91, p < 0.01). No other differences were found. There were no significant differences between the three treatment groups for mean scores for any of the answers on the questionnaire.

A total of 21 (12%) of the responders contacted health care providers because of recurrent symptoms and of these 17 (9.7% of responders) were given treatment. The treatments and symptoms are shown in Table 3. Eight women (4.5% of the 176 responders) reported that they had been prescribed antibiotics for recurrent symptoms within six weeks after their initial illness had abated. Five of these had previously been prescribed antibiotics at first contact with the clinic. A further 46 women (26% of the 176 responders) reported renewed symptoms from their breasts which they had managed themselves without contact with health care services. The mean number of days after recovery from the first episode that the new symptoms occurred was 16 (SD = 11.2). Twelve women (7 % of the 176 respondents) reported that they had stopped breastfeeding the baby from the breast subsequent to the episode of inflammation.

**Table 3:** Residual symptoms reported by women on the six week follow-up questionnaire (n = 176)

**Symptoms reported**	**Responders with symptoms** ***n *(%)**	**Responders with renewed symptoms who were given treatment** ***n *(%)**
Lumps in the breast	11 (6.3)	none
Redness of the breast	4 (2.3)	none
Breast tension	1 (0.6)	none
Breast boil	1 (0.6)	1 (0.6)
Itchy skin rash	1 (0.6)	none
Breast inflammation	48 (27.3)	16 (9.0)

**Treatments given**	***n***	
Antibiotics	8	
Acupuncture	7	
Lactation suppression medication	1	
Oxytocin nasal spray	1	There was no record of treatment given for 4 of the women who reported being treated

Table 4 shows the mean (SD) scores for all the questions on the follow-up questionnaire and comparison of means between favourable/less favourable outcome groups. The mean score for all responders for physical health (maximum 5) was 4.5 (SD = 0.6) and a total of four women reported that they still felt quite poorly. Regarding psychological well-being (maximum 5), the mean score was 4.4 (SD = 0.7). Four women reported that their psychological well-being was quite poor and one mother answered that her psychological well-being was very poor. There were no significant differences for assessments of health or psychological well-being between the groups: favourable/less favourable outcome, standard/higher education or primiparous/multiparous women. Those with recurrent symptoms (n = 67) showed significantly lower scores for perceptions of physical health (z = 3.7, p < 0.01), and psychological well-being (z = 2.1, p = 0.04) than those without recurrent symptoms (n = 109).

**Table 4:** Comparison of mean (SD) for items on the questionnaire: women's health, psychological well-being and perceptions of quality of care

	**All responders**	**Favourable outcome** ** (≤ 5 contact days)**	**Less favourable outcome** ** (≥ 6 contact days)**	**Z **^1^	***p***
**Item**	*n *= 176	*n *= 126	*n *= 50		
How I rate my physical health ^2^	4.5 (± 0.6)	4.5 (± 0.6)	4.4 (± 0.7)	0.45	0.67
How I rate my psychological well-being ^2^	*n *= 176	*n *= 126	*n *= 50		
	4.4 (± 0.7)	4.5 (± 0.7)	4.4 (± 0.8)	0.64	0.53
I was given best possible advice and instructions ^3^	*n *= 175	*n *= 125	*n *= 50		
	2.8 (± 0.4)	2.9 (± 0.3)	2.7 (± 0.4)	2.00	0.05
I had confidence in the midwife ^3^	*n *= 175	*n *= 126	*n *= 49		
	2.8 (± 0.4)	2.9 (± 0.4)	2.7 (± 0.5)	2.21	0.03
I had confidence in the doctor ^3^	*n *= 38	*n *= 15	*n *= 23		
	2.6 (± 0.6)	2.5 (± 0.6)	2.6 (± 0.7)	0.50	0.62

**I was given good information about:**					
How often I should breastfeed	*n *= 173	*n *= 124	*n *= 49		
Perceived Reality ^4^	3.1 (± 1.3)	3.0 (± 1.3)	3.3 (± 1.1)	1.67	0.10
Subjective Importance ^4^	2.7 (± 1.3)	2.6 (± 1.3)	3.0 (± 1.1)	2.24	0.03
Expressing milk by hand	*n *= 171	*n *= 123	*n *= 48		
Perceived Reality ^4^	2.9 (± 1.4)	2.9 (± 1.4)	3.0 (± 1.2)	0.40	0.70
Subjective Importance ^4^	2.2 (± 1.3)	2.2 (± 1.3)	2.3 (± 1.3)	0.63	0.53
Using a breast pump	*n *= 170	*n *= 123	*n *= 47		
Perceived Reality ^4^	3.4 (± 1.0)	3.4 (± 1.1)	3.6 (± 0.7)	0.60	0.60
Subjective Importance ^4^	3.0 (± 1.2)	2.9 (± 1.3)	3.3 (± 0.7)	1.45	0.15
Other ways of decreasing breast tension	*n *= 171	*n *= 123	*n *= 48		
Perceived Reality ^4^	2.4 (± 1.6)	2.4 (± 1.6)	2.4 (± 1.5)	0.38	0.70
Subjective Importance ^4^	2.4 (± 1.5)	2.4 (± 1.5)	2.5 (± 1.5)	0.54	0.60
					
How breast inflammation occurs	*n *= 173	*n *= 125	*n *= 48		
Perceived Reality ^4^	3.4 (± 0.9)	3.4 (± 0.9)	3.4 (± 0.9)	0.09	0.93
Subjective Importance ^5^	3.3 (± 0.9)	3.2 (± 0.9)	3.4 (± 0.9)	1.13	0.26
How to avoid overfull breasts	*n *= 173	*n *= 125	*n *= 48		
Perceived Reality ^5^	2.6 (± 1.4)	2.7 (± 1.4)	2.4 (± 1.4)	1.34	0.20
Subjective Importance ^4^	2.7 (± 1.4)	2.7 (± 1.4)	2.7 (± 1.5)	0.08	0.93

**I felt that the midwife:**					
Seemed to understand my situation	*n *= 172	*n *= 124	*n *= 48		
Perceived Reality ^4^	3.6 (± 0.7)	3.7 (± 0.6)	3.5 (± 0.8)	1.60	0.11
Subjective Importance ^4^	3.6 (± 0.6)	3.6 (± 0.7)	3.7 (± 0.6)	1.36	0.18
Showed that she cared about me	*n *= 171	*n *= 123	*n *= 48		
Perceived Reality ^4^	3.8 (± 0.5)	3.8 (± 0.5)	3.7 (± 0.6)	0.10	0.32
Subjective Importance ^4^	3.8 (± 0.5)	3.7 (± 0.5)	3.8 (± 0.5)	0.52	0.60
Supported me in my role as a new mother	*n *= 171	*n *= 123	*n = *48		
Perceived Reality ^4^	3.3 (± 1.2)	3.3 (± 1.2)	3.3 (± 1.2)	0.07	0.95
Subjective Importance ^4^	3.3 (± 1.2)	3.2 (± 1.2)	3.4 (± 1.1)	1.0	0.32

**I was given enough opportunity to talk to the midwife about:**					
My health in general	*n *= 170	*n *= 124	*n *= 46		
Perceived Reality ^4^	3.3 (± 1.1)	3.3 (± 1.2)	3.4 (± 1.0)	0.05	1.0
Subjective Importance ^4^	3.1 (± 1.2)	3.1 (± 1.2)	3.3 (± 1.0)	1.06	0.30
My breast inflammation	*n *= 167	*n *= 121	*n *= 46		
Perceived Reality ^4^	3.8 (± 0.6)	3.8 (± 0.6)	3.7 (± 0.6)	0.81	0.42
Subjective Importance ^4^	3.6 (± 0.7)	3.6 (± 0.8)	3.7 (± 0.5)	0.13	0.90

^1 ^= Mann Whitney U-test; ^2 ^= range 1–5; ^3 ^= range 1–3; ^4 ^= range 1–4

The women's perceptions of the understanding manner, the respect and support given by the midwives showed high scores as did the subjective importance of these aspects. Scores showed also that they felt they were given enough opportunity to talk about their health and their breast problems. Scores were generally lower for the perceived reality and subjective importance of information about hand expression, other means of decreasing their increased breast tension and avoidance of overfull breasts.

Scores for being given the best possible advice and instructions and for confidence in midwives and doctors were generally high. However the less favourable outcome group (≥6 contact days) had significantly lower scores than the favourable outcome group (≤5 contact days) for being given the best possible advice and instructions (p = 0.05). Women with a less favourable outcome had also lower mean scores for confidence in the midwives (p = 0.03) and higher mean scores for the subjective importance of information about how often breastfeeding should occur than women with a favourable outcome (p = 0.03).

Multiparous women showed higher mean scores for perceived reality of information about different methods to relieve breast tension than primiparous women (z = 2.1, p = 0.03). Women with standard education level had significantly higher scores than women educated to college/university level for the subjective importance of information about how often breastfeeding should occur (z = 2.8, p < 0.01) and how to avoid overfull breasts (z = 2.2, p = 0.03). They had also higher scores for the perceived reality of being supported in their role as a new mother (z = 2.7, p < 0.01) and the subjective importance of this support (z = 2.7, p < 0.01). There were no significant differences between those with or without reoccurrence of symptoms for mean scores for any of the 17 items.

## Discussion

This study was carried out at one breastfeeding clinic in southern Sweden and because of the limited uptake area the results may not be applicable to every population. The use of six weeks as the point at which the follow-up was carried out was arbitrary and it is possible that some women may have had recurring symptoms beyond the six week period. Further research should include inter-rater reliability testing of the scales used for measurement of symptoms, in order to judge the usefulness of the scales.

This study confirms the findings of other researchers [2,16] that symptoms of breast inflammation most often appear in the early postpartum weeks. Reports of bilateral symptoms have varied between 3% [16] and 37% [10] and our finding was that 14% had bilateral symptoms. This difference may be to some extent caused by the different sizes of the study populations. Use of nipple shields and pacifiers was recorded since it has been suggested by others that their use is associated with breastfeeding problems [21,22]. Both damaged nipples and the use of a nipple shield increased the risk of a less favourable outcome. It is likely that women in this study were using nipple shields because of persistent damaged nipples. Other researchers have found damaged nipples to be related to the occurrence of lactation mastitis [9,23] and some have cited damaged nipples as the route for bacterial invasion leading to mastitis [23,24]. It was seen that 63% of the women in this study had no nipple problems. However, those with nipple damage needed longer contact with the breastfeeding clinic.

Osterman & Rahm [2] stipulated a temperature of over 38°C as an inclusion criterion for their study whereas there was no stipulation in our study. Since mastitis is an inflammation, which may or may not be accompanied by infection [6] it seemed judicious to also include those without fever. More than half of women in this study for whom temperature was recorded (52%) had severe fever with temperatures as high as 40.7°C but it was seen that the presence or absence of fever at first contact did not affect outcome measured by number of contact days. It has been suggested earlier that clinical signs and symptoms may not aid the clinician in deciding which women require antibiotic treatment [12] and results from the present study support this finding. The problem of international non-consensus on the definition of mastitis has been commented on earlier [5]. International collaboration in future studies, rather than each researcher drawing up her/his own inclusion/exclusion criterion, might take research into lactation mastitis forward at a quicker pace. In order to facilitate comparison of results from international studies, it would be advantageous if scales for the measurement of symptoms of breast inflammation could be agreed upon as a standard for the reporting of mastitis.

The surprisingly low prescription of antibiotics in this study has been reported and discussed in the RCT [12] but is nevertheless worthy of some attention here. Reports from Australia, New Zealand, USA and Canada have shown that between 77% and 97% of women are prescribed antibiotics [16,23-26]. In the RCT in which this study was nested, 15% (31 of 210) of women were initially prescribed antibiotics. In Wambach's study 97% (30 of 31) women were prescribed antibiotics and a 19% recurrence of symptoms was reported by the six week follow-up [16]. It was seen in this study that 21 (12%) of responders contacted health care services because of recurrent symptoms; 8 of them were prescribed antibiotics and for 5 of them, this was the second prescription of antibiotics. A further 46 women (26%) reported renewed symptoms which they had managed themselves without recourse to new contact with health care services. This may indicate that the symptoms were mild. The fact that 3.3% of the cohort developed breast abscess during the study period may be compared to findings from Australia where the incidence was 3.0% and the prescription of antibiotics considerably higher [27]. None of the women in Wambach's study developed a breast abscess but the differences in sample sizes makes comparison with our study difficult [16].

Ninety-six percent of the women reported that their physical health was "very good" or "quite good" and this finding is similar to the 91% reported in an earlier Swedish study [28] of postpartum women's health. A total of five women (3%) felt their psychological well-being to be poor, which is a smaller proportion than in a recent study of breastfeeding women in Australia where Henderson et al reported an 18 % incidence of poor psychological health [29]. The remaining 97% answered that their psychological well-being was good or very good. Conversely, Groer showed that breastfeeding was somewhat protective of negative moods and stress [30]. Results of this study may support Groer's findings and to some extent explain the ability of new mothers to withstand the burden of inflammatory symptoms of the breast whilst having a baby to care for as has previously been described [8]. Even though a large proportion of women with recurring symptoms had managed these themselves without recourse to the breastfeeding clinic or other health care services, they rated their physical health and psychological well-being lower than those without symptoms. This is indicative of the impact that inflammatory breast symptoms can have on women's health during a period when the demands of caring for small children are high. Women made spontaneous reports of stopping breastfeeding; this information was not actively sought, which is a weakness in the study design.

In this study the mothers generally gave higher scores for the way in which they were met and supported by the midwives than for the advice given to them about how to manage their breast symptoms, for both perceived reality and subjective importance. This is an interesting finding although difficult to interpret. It may simply be that the women expected to be given good care advice whereas an empathetic meeting with the midwife was something they could not take for granted. It is also interesting that those with a less favourable outcome were less sure about the quality of the advice and instructions given to them and expressed a lack of confidence in the midwives. This poses the question whether the lack of confidence occurred because the illness was protracted or whether the illness was protracted because of non-adherence to suggested care regimes because of lack of confidence. Women may have their own ideas about what is the best way to tackle their problem and may experience a lack of confidence if the midwife's advice does not coincide with these ideas. More knowledge would have been gained if follow-up questions had been asked to ascertain why some women didn't feel they had been given the best possible help. Further research could address the question of women's adherence to suggested care regimes.

Women whose illness was protracted and women who had standard education gave higher scores to the subjective importance of information about how often they should feed their baby. This is somewhat surprising since the infant's free access to the breast is part of all breastfeeding information in Sweden. Breastfeeding is very time consuming and it may be that some women were reluctant to allow breastfeeding to take precedence over all other activities. The episode of breast inflammation may have made them more receptive to the information offered.

The fact that women with standard education perceived more support for their role as a new mother and felt this to be of greater subjective importance than women with higher education may be a sign that the midwives offer individualised care. In their contacts with new mothers the midwives may have learnt which of them have the potential to access the information they need and therefore concentrate their efforts on those in more need of support.

## Conclusion

Women whose breast inflammation is accompanied by damaged nipples may require more vigilant follow-up than those without. Initial fever may not be indicative of outcome and treatment by antibiotic therapy for breast inflammation may be necessary less often than has been supposed. International research collaboration might help us find the optimal level of antibiotic therapy for this group of women. This is an important consideration for the global community. Scales for the measurement of symptoms should be further developed. The relationship between symptom persistence and confidence in the midwives and in care received is unclear.

## Competing interests

The author(s) declare that they have no competing interests.

## Authors' contributions

LJK, MLH-L and BWL designed the study. LJK carried out the data collection for the questionnaire and entered the data onto the computer. LJK, MLH-L and BWL carried out the statistical analyses. LJK drafted the manuscript, which was adjusted after discussions with MLH-L and BWL.
